# Transorbital Neuroendoscopic Approaches: Historical and Tactical Review of Orbital Corridors: A Scoping Review

**DOI:** 10.1055/a-2525-5851

**Published:** 2025-02-24

**Authors:** Rose Fluss, Muhammed Amir Essibayi, KiChang Kang, Daniel Popoola, Dileep D. Monie, Patrick Colley, Vijay Agarwal

**Affiliations:** 1 Department of Neurological Surgery, Montefiore Medical Center, Leo M. Davidoff, Bronx, New York, United States

**Keywords:** endoscopic neurosurgery, minimally invasive, transorbital approach, neurosurgery

## Abstract

**Introduction:**

The orbit is a useful corridor underutilized by the neurosurgical community. The aim of this manuscript is to describe this well-established approach in the neurosurgeon's tool box.

**Methods:**

A scoping review of 363 articles containing transorbital neuroendoscopic surgical approaches were reviewed by two independent reviewers for inclusion in this report.

**Results:**

Discussed here are the four transcutaneous transorbital approaches, including the superior eyelid crease (SLC), and upper eyelid approaches, lateral (retrocanthal) transorbital approach, inferior (preseptal) transconjunctival approach, and the medial (transcaruncular) orbitotomy approach.

**Conclusion:**

This practical review will highlight the surgical approaches, historical origins, indications, and contraindications of all the orbital corridors to the cranium.

## Introduction

Transorbital neuroendoscopic surgery (TONES) has emerged as a minimally invasive approach for accessing the intracranium. By utilizing either a transorbital or transcutaneous entry point, surgeons can perform open or endoscope-assisted procedures on various skull base and intraorbital pathologies. The evolution of both techniques and technology has allowed the expansion of indications from initially orbital lesions to now include tumors, vascular lesions, and other pathologies throughout the anterior and middle cranial skull base.

Transcutaneous transorbital approaches, including the suprabrow, superior eyelid crease (SLC), and upper eyelid approaches, utilize small incisions and dissection planes superficial to the periorbita for bone flap elevation of the orbital roof or lateral wall. Transconjunctival transorbital techniques, such as the preseptal, retroseptal, and transcutaneous transconjunctival approaches, allow access by discretely progressing between the conjunctiva and orbital septum.

## Methods

### Search Strategy

**Fig. 1:**
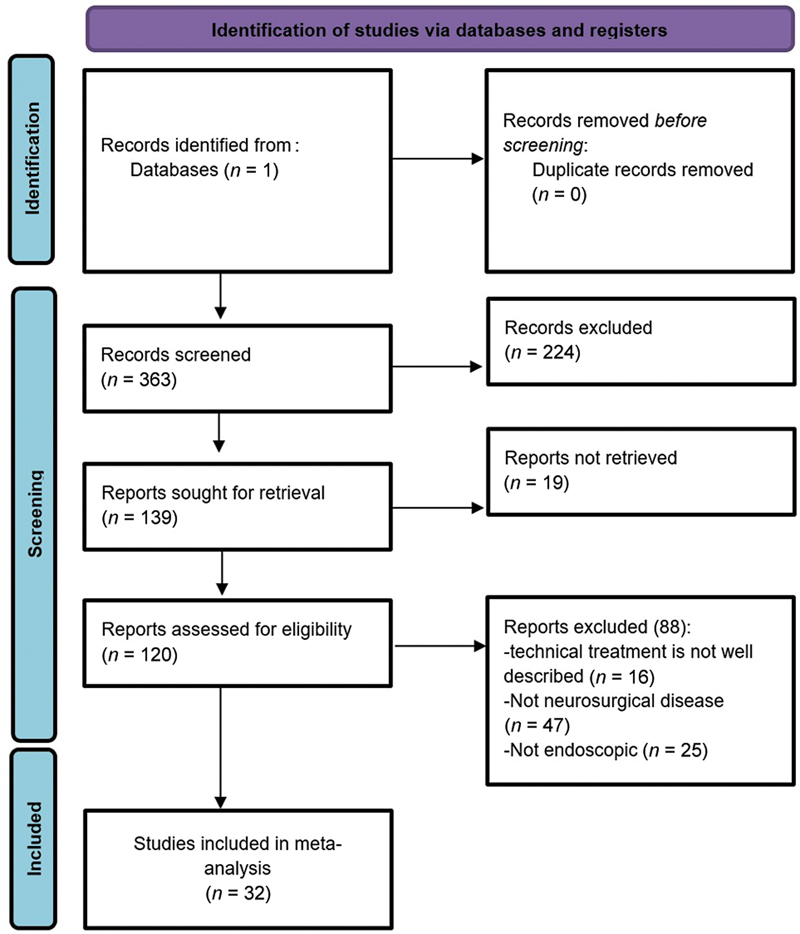
Flow diagram of study review and inclusion process.

A comprehensive literature search was conducted on PubMed database on January 14, 2024, to identify relevant articles describing transorbital neuroendoscopic surgical approaches. The following search terms and Medical Subject Headings (MeSH) were used in various combinations: “transorbital,” “superior eyelid crease,” “orbitotomy,” “transconjunctival,” “retrocanthal,” “transcaruncular,” “neuroendoscopic surgery,” “TONES,” “minimally invasive,” “skull base.” The search was limited to articles published in the English language. Studies were included if they met the following criteria: (1) Described surgical techniques, indications, contraindications, or complications of transcutaneous or transconjunctival transorbital neuroendoscopic approaches to the skull base; (2) included human subjects or cadaveric studies; (3) provided detailed descriptions of the surgical procedure and anatomical considerations; and (4) published in peer-reviewed journals. Articles were excluded if they: (1) focused solely on non-endoscopic transorbital approaches; (2) did not provide sufficient details on the surgical technique or anatomical considerations; and (3) were case reports, editorials, or conference abstracts. A flow diagram of the study review and inclusion process is illustrated in Fig. 1 .

### Data Extraction and Analysis

Two independent reviewers screened the titles and abstracts of the retrieved articles ( *n* = 363) based on the inclusion and exclusion criteria. Full-text articles of the selected studies were then reviewed for eligibility. Any discrepancies were resolved through discussion and consensus. Data extracted from the included studies encompassed surgical techniques, indications, contraindications, complications, and outcomes. A narrative synthesis of the extracted data was performed, focusing on the key aspects of each transorbital neuroendoscopic surgical approach.

## Superior Eyelid Crease Approach

**Fig. 2:**
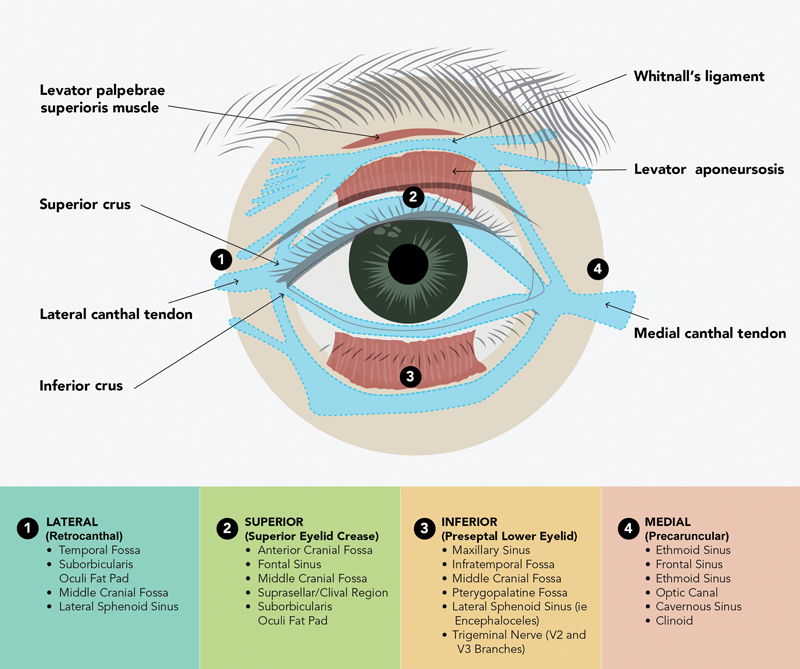
Summary of pathologies reached by each orbital approach. Encountered ligaments transposed over each corridor.

The superior eyelid crease has long-standing roots in oculoplastics, being commonly used for blepharoplasties and in orbital decompression for Graves disease. However, for the neurosurgeon this corridor is a relatively new venture in addressing the lateral and superolateral walls of the orbit. Traditionally, just an upper eyelid incision is made where the scar hides in the natural lid crease. The neurosurgeon can gain exposure that traditionally required a fronto-orbital, pterional, or orbitozygomatic approach ( Fig. 2 ). We discuss here the superior eyelid crease (SLC) approach, operative considerations, as well as the optimal surgical corridors.

The case usually begins with evaluating a preoperative computed tomography (CT) scan to evaluate the lateral extent of the frontal sinus. 1 2 This approach becomes relatively contraindicated if a sinus breach and subsequent infection is likely. Caution is then taken in tacking the upper and lower eyelids together with suture to avoid corneal abrasion. 3

An incision is made in the supratarsal fold, roughly 3 to 6 mm lateral to the medial canthus. The incision can be safely extended 2.5 cm past the lateral canthus, taking care to curve slightly upwards to avoid the facial nerve (CN VII). 4 The orbicularis oculi muscle is then encountered, and a plane is created until orbital septum is visualized. The orbital septum is divided from the orbital roof and one can follow the corridor below the orbital roof taking great care not to violate the periorbita.

Once the periosteum is reached it is sharply incised to reveal the orbital rim. 5 The bone of the orbital rim is exposed until the supraorbital notch is reached. Care is taken to free up the supraorbital nerve from the supraorbital notch using sharp dissection. The nerve is then reflected laterally for its protection. The orbital rim and frontal bone are then in view. Temporalis muscle fascia is encountered and incised if more lateral exposure is needed. A craniotomy removing the orbital rim can then be performed.

### Indications

Craniotomies performed through an SLC exposure are particularly well-suited for treating anterior cranial fossa lesions, encompassing olfactory groove, planum sphenoidale, and sphenoid wing meningiomas, especially when their dimensions remain within the order of 3 cm or less in the largest diameter. Furthermore, it has proven its efficacy in the management of sellar and parasellar lesions, including pituitary adenomas, craniopharyngiomas, and tuberculum sellae meningiomas. This approach also extends its utility to the treatment of intra-axial lesions situated in the orbital gyrus, medial orbital gyrus, rectus gyrus, and frontal pole, which can encompass pathologies like cavernomas and gliomas. 1 Endoscopic-assisted technique can be used for wider visualization of the sellar and parasellar regions, as well as the upper third of the clivus, anterior third ventricle, and interpeduncular cistern. 2

**Fig. 3:**
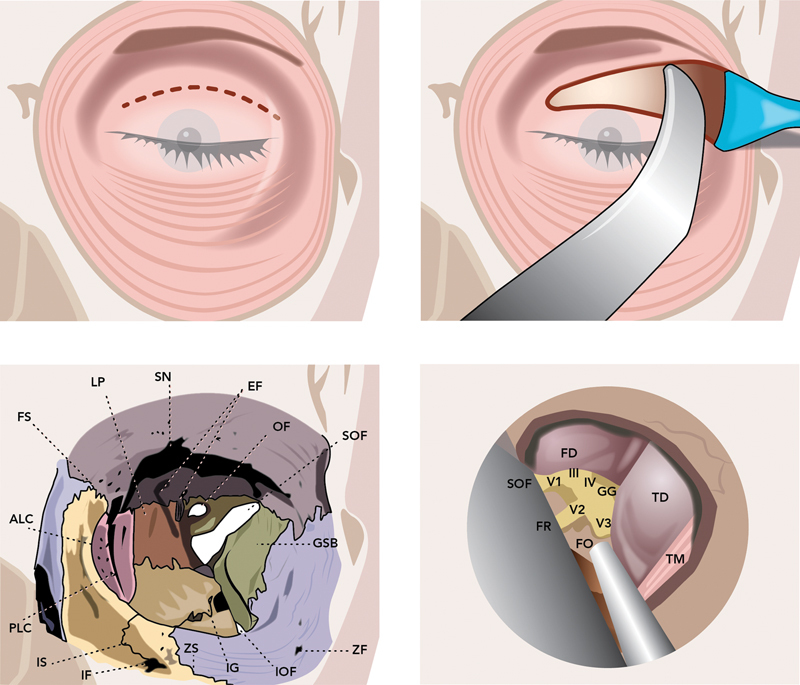
Key landmarks are identified. ALC, anterior lachrymal crest; EF, ethmoidal foramen; FD, frontal dura; FO, foramen ovale; FR, foramen rotundum; FS, frontomaxillary suture; GG, gasserian ganglion; GSB, greater wing of sphenoid bone; IF, infraorbital foramen; IG, infraorbital groove; III, CN III (oculomotor nerve); IOF, inferior orbital fissure; IS, infraorbital suture; IV, CN IV (trochlear nerve); LP, lamina papyracea; OF, optic foramen; PLC, posterior lachrymal crest; SN, supraorbital notch; SOF, superior orbital fissure; TD, temporal dura; TM, temporalis muscle; V1, ophthalmic branch of CN V (trigeminal nerve); V2, maxillary branch of CN V; V3, mandibular branch of CN V; ZF, zygomaticofacial foramen; ZS, zygomaticomaxillary suture.

The SLC approach has been described as applicable for unruptured anterior communicating artery (ACoA) aneurysms and aneurysms located in the C7 segment of the internal carotid artery (ICA). This approach provides direct access to essential neuroanatomical structures, including the ipsilateral and contralateral optic nerve (CN II), the ipsilateral oculomotor nerve (CN III), the branches of the ipsilateral trigeminal nerve (CN V), and the interpeduncular cistern ( Fig. 3 ). By skillfully opening the proximal sylvian fissure, it further extends its reach to the medial carotid artery complex and the medial temporal lobe.

### Contraindications and Complications

This approach is not advisable for lesions that extensively involve the middle fossa or cavernous sinus, as it may limit the surgeon's ability to access and manage such complex anatomical regions effectively. Additionally, lesions located in the superior and middle gyrus of the frontal lobe and the precentral gyrus pose challenges when approached from this angle, potentially hindering precise surgical intervention. 2

Furthermore, tumors that are significantly adherent to vascular structures are not well-suited for the superior eyelid crease approach due to the heightened risk of bleeding and the inherent difficulty in achieving proximal vascular control. Lastly, while not an absolute contraindication, the presence of significant edema and associated hydrocephalus is considered a relative contraindication, warranting careful evaluation and consideration before opting for this surgical approach. 4

## Lateral (Retrocanthal) Transorbital Approach

In 1889, Kronlein first described the lateral approach (LA) to orbital craniotomy, which was designed and often used to access periorbital and intraconal lesions located dorsal, basal, or lateral to the optic nerve. 6 Its application has since expanded to accessing the cavernous sinus 7 and intracranial temporal structures including the hippocampus, amygdala, even as far as the cerebral peduncle. 8

According to Abou-Al-Shaar and colleagues, 6 the traditional LA approach requires a canthotomy followed by a dissection down to the lateral orbital rim. The periosteum is then dissected to mobilize the temporalis muscle, exposing the lateral orbital wall. Next, orbitotomy is completed by cutting along the frontozygomatic suture superiorly and zygoma inferiorly to extract a bone flap of the lateral rim, which can be secured when done with titanium plates. This exposes the greater wing of the sphenoid bone which can be further drilled to access the intra-, peri-, and retro-orbital structures such as the cavernous sinus and anterior clinoid process. Variations of the LA include the total lateral orbitotomy (TLO) and the modified lateral orbitotomy (MLO) which primarily differ in the size of the extracted lateral orbital wall bone flap, with the TLO being larger than the MLO. A third is the endoscopic variation.

The TLO creates a bone flap that spans the superior orbital notch through the inferior orbital notch, encompassing the lateral wall of the orbit. 9 The TLO grants the surgeon relatively better access to the orbital apex to manage deep orbital lesions with additional access to the anterior cranial fossa, if necessary. However, the TLO may endanger important neurovascular structures coursing within the lateral orbital space including the supraorbital artery, superior ophthalmic vein, posterior ethmoid artery, and nasociliary nerve, requiring extra caution to protect these structures. 9 On the other hand, the MLO extracts a smaller bone flap bordered by the superior and inferior orbital fissures. 10 The intended benefit of the MLO is the ability to reflect rather than transect this dural covering to reveal the cavernous sinus for addressing cavernous sinus lesions. The MLO also provides access to the sphenoid section of the sylvian fissure, opticocarotid cistern, and anterior clinoid process. 6 Due to its relatively less invasive access to the cavernous sinus, some institutions use the MLO to biopsy or resect small anterior cavernous sinus lesions. 6 However, the MLO's smaller cranial access window benefit also costs the surgeon maneuverability.

The endoscopic variation of the LA was first described using computer models in 2012 and subsequently using cadavers in 2014 before the first clinical case report of its clinical application was published in 2015. 7 8 Unlike the TLO and MLO, the endoscopic approach does not produce a replaceable bone flap. Rather, the orbit is accessed with an endoscope passing through a retrocanthal skin incision, the orbicularis oculi muscle fibers, and the periosteal layer. Then the inferior and superior orbital fissures are exposed and a keyhole orbitotomy through the greater wing of the sphenoid exposes the dura just overlying the temporal pole. Through this window, one group accessed the hippocampus, amygdala, and the lateral aspect of the cerebral peduncle in cadavers, 8 while the second group accessed the cavernous sinus. 7

### Indications

Existing evidence suggests the LA may be suitable for surgical targets deep within the temporal lobe as well as the cavernous sinus, middle fossa floor, and the Meckel cave region. 6 7 11 Especially for cavernous sinus pathologies, this approach, of every other transorbital approach, provides the best view and working angle for the surgeon with a direct view of the sinus. The endoscopic LA is additionally useful for amygdalohippocampectomy as previously demonstrated in a clinical case of hippocampal sclerosis secondary to gliosis, and for resecting lesions within the entorhinal cortex and its subjacent white matter. 11 The LA promises certain advantages that boost its suitability for certain skull base neurosurgical procedures. The first is proximity advantage as the access point for the LA approach is reportedly closer to sellar and parasellar structures compared with other surgical approaches including the intranasal approach. 12 Especially when compared with traditional craniotomies, this proximity advantage possibly diminishes unnecessarily manipulating cortical structures along the path to the surgeon's skull base target. This minimizes unnecessary iatrogenic injuries and the resultant postoperative neurological deficits such as hemiparesis, vision loss, and language and memory impairment that are commonly reported in traditional craniotomies. Second, the LA provides a better access and viewing angle to the cavernous sinus when compared with a similar less-invasive alternative endonasal approach. 13 In fact, in a case report, the endoscopic transorbital LA was required as a complement to the endoscopic endonasal approach for resection of parts of a pituitary adenoma in the cavernous sinus that were inaccessible by the endonasal approach. 13 This strengthens the argument for the LA's suitability for cavernous sinus pathologies. Third, the LA, especially the endoscopic approach, boasts of a cosmetic advantage over traditional craniotomies with the relatively smaller incision. Furthermore, LA patients reportedly have a shorter length of hospital stay that arguably reduces the cost of treatment compared with the traditional temporal approach. However, given the risks of damage to the globe, complications such as orbital pseudomeningocele have limited cases.

## Inferior (Preseptal) Transconjunctival Approach

Since its earliest description in 1924, the preseptal transconjunctival approach has gained significant popularity for orbital floor reconstruction and inferomedial orbital wall surgery, primarily due to its inherent advantages over transcutaneous techniques. 14 This surgical approach entails a meticulous incision within the lower conjunctival fornix, positioned just superior to the orbital septum while preserving the integrity of orbital fat. By maintaining these structural components, the risk of lower eyelid complications is considerably minimized. If deemed necessary, this incision can be extended through a lateral canthotomy to enhance exposure of the lateral orbital wall. The subsequent blunt dissection in the avascular plane, positioned deep to the orbicularis oculi and superficial to the septum, facilitates access to the intraconal space. Moreover, the utilization of an endoscope for direct endoscopic visualization of surgical anatomy and the execution of bone work and fracture repair further enhances precision. 6 15

This approach offers an expansive view of inferior orbital structures, encompassing the orbital floor, infraorbital rim, and the maxillary sinus wall, all through a concealed transconjunctival incision. Additionally, it allows for ready accessibility to the inferomedial, basal, and lateral intraconal compartments, thus facilitating the reconstruction of isolated or combined fractures in these regions. 16 17 18 Importantly, as this approach does not necessitate a skin incision or the disruption of the orbital septum, it drastically reduces the risk of postoperative lower eyelid complications, such as retraction and ectropion, when compared with transcutaneous techniques.

The minimal lateral scar achieved through this technique yields superior cosmetic outcomes, which are of paramount importance in the periorbital region. 15 The preservation of orbital fat and septum architecture significantly mitigates the risk of postoperative enophthalmos. When complemented by rigid fixation using miniplates, this approach empowers surgeons to achieve anatomically precise reconstructions of the orbital floor and walls.

### Indications

Given its minimally invasive nature, the preseptal transconjunctival approach is ideally suited for the reconstruction of isolated orbital floor fractures and combined fractures involving the inferior orbital walls. This technique boasts a distinct advantage in that it eliminates the need for skin incisions or orbital septum disruption, thereby reducing the risk of postoperative lower eyelid complications when compared with external approaches. 6 Furthermore, it provides ample exposure for the placement of reconstructive materials such as miniplates while safeguarding the delicate anatomical relationships of periorbital structures. 6 15 The discrete nature of the conjunctival incision results in optimal cosmetic outcomes. In the hands of experienced surgeons, the preseptal transconjunctival approach offers a plethora of advantages for the surgical management of specific inferior orbital pathologies. With minimal risk of lower eyelid or enophthalmos complications, coupled with optimal cosmesis and the benefits of direct endoscopic visualization, it stands as a viable alternative to open techniques for carefully selected cases. Ongoing experience with this approach holds the potential to solidify its role as the preferred technique in appropriately selected cases.

### Contraindications and Complications

Relative contraindications for this approach primarily include cases involving large tumors of the inferior orbit, where the limited corridor may impede complete excision. 6 14 Lesions situated superiorly or medially that cannot be adequately accessed via an inferior transconjunctival route may be better suited for treatment through an external approach. Additionally, due to the technical complexity of the technique, it is best undertaken by experienced surgeons who can ensure the preservation of critical anatomical landmarks. In instances where extensive bone resection is required or if the operator lacks the necessary experience, alternative techniques should be contemplated. Although technically more demanding than open approaches due to the constraints of the working corridor, satisfactory exposure can be achieved with accumulated proficiency. This approach may not be suitable for managing large infraorbital tumors or lesions in superior orbital locations that are inaccessible via an inferior transconjunctival incision. It is important to note that an additional lateral canthotomy, while enhancing exposure in select cases involving the lateral wall, adds complexity and increases operative time. 6 16

## Medial (Transcaruncular) Orbitotomy

The transcaruncular medial orbitotomy is an incision through the medial canthal region that provides access to the medial orbital wall and apex. 19 The approach begins with an incision through the medial conjunctiva between the caruncle and plica semilunaris, extending approximately 8 to 10 mm superiorly and inferiorly from the caruncle. 20 The incision transects the lateral quarter of the caruncular–conjunctival junction. After the incision is made, blunt dissection is performed posteriorly toward the posterior lacrimal crest using scissors. The posterior lacrimal crest is palpated, and the dissection plane is established between it and the orbital septum, avoiding disruption of the medial canthal tendon and lacrimal apparatus.

Once the periosteum behind the posterior lacrimal crest is exposed, it is widely opened using cautery and an elevator. The periosteum is elevated superiorly and inferiorly off the medial orbital wall to gain access to the apex and body of the orbit in the subperiosteal plane. 21 22 Osteotomy of the lamina papyracea can be performed with rongeurs or a high-speed drill, depending on the desired exposure. The anterior and posterior ethmoidal arteries may be identified and spared or cauterized depending on the needed exposure. Gentle retraction of the orbital contents with a malleable retractor allows wide exposure of the medial orbit for procedures such as fracture repair, biopsy, or decompression. 23 Finally, the caruncle and conjunctiva are re-approximated and closed with absorbable sutures.

### Indications

The transcaruncular medial orbitotomy is commonly used for a variety of indications given its excellent exposure of the medial orbit. It can be utilized for the repair of isolated medial orbital wall fractures that result in enophthalmos or muscle entrapment. 9 This approach also enables biopsy or excision of various medial intraconal lesions such as cavernous hemangiomas or pleomorphic adenomas of the lacrimal gland. Decompression of the bony orbit via the medial wall is performed for Graves orbitopathy to improve proptosis or optic neuropathy. 24 Drainage of orbital abscesses that occur from extension of sinusitis is facilitated by the transcaruncular approach. 20 Lesions of the orbital apex including meningiomas, hemangiopericytomas, and metastatic tumors can also be accessed for biopsy or resection. 25 Optic canal decompression for traumatic optic neuropathy can be achieved as well through this exposure. 26 Medial blowout fractures causing rectus muscle entrapment or implant extrusion are managed through the transcaruncular approach. 9 Finally, this technique is ideal for marsupialization of frontoethmoid mucoceles and excision of the cyst lining. 20

### Contraindications and Complications

This approach is generally contraindicated in patients with extensive lacrimal system scarring from previous surgery or trauma, which increase the risk of inadvertent damage to the nasolacrimal drainage structures. 24 27 Furthermore, prior radiation to the medial canthus region is a possible contraindication because it can lead to increased fibrosis and poor wound healing. 28 29 In cases with tumor invasion into the caruncle or medial canthal tendon, obtaining tumor-free margins might not be feasible. 28 Severe medial canthal dystopia or lid malposition may also make safe surgical access very difficult. 29 30 Potential intraoperative complications include damage to the nasolacrimal system, disruption of the medial canthal tendon, and injury to the medial rectus muscle or surrounding orbital structures. 31

## Conclusion

This article illustrates that transorbital endoscopic surgery has rapidly developed into a key technique for minimally disruptive access to the anterior and middle cranial base. Both transcutaneous and transconjunctival approaches offer discreet incisions for excellent cosmesis. The transcutaneous subgroup provides versatile bony exposure through orbital osteotomies, while transconjunctival techniques maintain integral soft tissue planes. Angled endoscopes through these corridors offer panoramic visualization of deep skull base targets.

Outcomes studies have shown feasibility and safety across a range of pathologies, from craniofacial fractures to tumors and vascular lesions. Complication rates are favorable but technical learning curves exist. Ongoing developments in three-dimensional printing, augmented reality, and robotics promise to further enhance capabilities. Although certain limitations exist regarding lesion size and lateral extent, TONES has proven a versatile addition to the armamentarium of skull base surgeons. Continued advances will enhance its role for anterior and middle fossa access with minimal cosmetic and cortical disruption.
